# Metabolomics study of dried ginger extract on serum and urine in blood stasis rats based on UPLC‐Q‐TOF/MS

**DOI:** 10.1002/fsn3.1929

**Published:** 2020-10-05

**Authors:** Min Su, Gang Cao, Xiaoli Wang, Raftery Daniel, Yan Hong, Yanquan Han

**Affiliations:** ^1^ Grade Three‐level Laboratory of TCM Preparation State Administration of Traditional Chinese Medicine/Anhui Province Key Laboratory of Chinese Medicinal Formula (Anhui University of Chinese Medicine)/Engineering Technology Research Center of Modernized Pharmaceutics The First Affiliated Hospital of Anhui University of Chinese Medicine Hefei China; ^2^ Anhui University of Chinese Medicine Hefei China; ^3^ Zhejiang Chinese Medical University Hangzhou China; ^4^ Northwest Metabolomics Research Center/Mitochondria and Metabolism Center Department of Anesthesiology & Pain Medicine University of Washington Seattle WA USA

**Keywords:** blood stasis syndrome, dried ginger, metabolomics, MS, UPLC‐Q‐TOF

## Abstract

Blood stasis syndrome (BSS) is the pathological basis of many cardiovascular diseases. Ginger is often used as herbal medicine, condiment, and health food in China and Southeast Asia to improve some symptoms of cardiovascular disease, but its mechanism of efficacy and metabolic processes is not clear enough. In this study, a rat model of BSS was successfully established and treated with different doses of dried ginger extract. After the end of the administration period, the blood and urine of 5 groups of rats were collected for metabonomic analysis. Multivariate statistical analysis was used to explore metabolites and metabolic pathways, and the correlation between metabolites and pharmacodynamic indicators was further explored. The experimental results show that the pharmacodynamic indicators of dried ginger group (DG) extracts of different doses have different degrees of changes than model group (MG), and the high dose of dried ginger group (GJH) changes is the most significant (*p* < .05 or *p* < .01). Besides, 22 different metabolites were identified in the experiment. These metabolites mainly involve seven metabolism pathways in different impact value. DG has therapeutic effects on BSS rats by regulating multiple metabolic pathways. This study provides an effective method for understanding the metabolic mechanism of DG extracts on BSS.

## Graphical abstract

1. To study the effect of dried ginger on blood stasis syndrome from the perspective of metabolomics.
2. Three metabolic pathways are the most critical ways for dry ginger to act on blood stasis syndrome.
3. The correlation between biomarkers illustrates the dose effect.

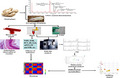

## 1 INTRODUCTION

Cardiovascular disease is one of the leading causes of death worldwide, affecting not only high‐income but also low‐ and middle‐income countries. Nearly 80 percent of all estimated cardiovascular disease‐related deaths worldwide now occur in low‐ and middle‐income countries, where nearly 30 percent of all deaths are attributable to cardiovascular disease (Kelly et al., 2012). BSS is a state of poor blood circulation due to increased blood viscosity and concentration, which is closely related to many diseases, such as coronary heart disease, infertility, and cancer (Xiao‐yan et al., 2018). It is a complex pathological system, usually accompanied by vascular endothelial injury, inflammation, liver and kidney injury, and other symptoms, which further promote the occurrence of blood stasis(Liu et al., 2017; Ma et al., 2009). In modern medicine, the cognition of BSS mainly refers to insufficient blood circulation, decreased blood flow to the body, or impurities in the blood (Jian et al., 2012). The pathological indexes of BSS include hemorheology, coagulation function (Cao et al., 2018). The perceptions of BSS are changing, as well as its management. Lifestyle factors involving diet now feature prominently as the more important contributors to the pathogenesis of BSS than genetics, as a result, TCM and healthy food have received extensive attention in the treatment of BSS due to their natural nature.

Ginger (Zingiber officinale Rosc.) is taxonomically characterized as perennial, aromatic, tuberous, and nontuberous rhizomes (Jatoi et al., 2007). Mainly produced in China, India, and other places and used as food seasoning ingredients and medicinal resources, it is rich in phytochemicals, such as gingerols, shogaols, and zingerone (Brahe et al., 2016). In recent years, ginger has attracted increasing attention due to its pharmacological properties, such as its anti‐inflammatory, anticarcinogenic, and antioxidant activities (Ezzat et al., 2018; Lee et al., 2008, 2015), especially for the treatment of diseases related to inflammation, oxidative stress, and cardiovascular diseases (Gunathilake & Rupasinghe, 2015; Peng et al., 2012; HASANAIN et al., 2016; Singh et al., 2008; Tohma et al., 2017). In China, Ginger is a commonly used medicinal and food dual‐use traditional Chinese medicine, it has a long history of medicinal use and significant clinical efficacy, and it is first published in Shennong Bencao Jing and has a history of more than 2,000 years (Fang et al., 2017). The main function of ginger is to treat colds, dispel cold, warm the stomach, and stop vomiting. The results of pharmacological studies have confirmed that ginger has the effects of vomiting, strengthening the heart, and anti‐inflammatory. It has a good therapeutic effect on cold and coughing, and has a certain preventive effect on the occurrence of cardiovascular, cerebrovascular diseases, and tumors. Generally, dried ginger is obtained by drying fresh ginger at low temperature. Due to the heating and drying process, dried ginger and fresh ginger have some changes in the content and composition of chemical components. The efficacy of dried ginger is mainly warming blood and promoting blood circulation, warming the lungs, relieving cough and vomiting, and treating abdominal pain, etc. Pharmacological research shows that DG has effects such as improving cardiovascular function, protecting liver and choleretic anti‐ulcer, anti‐inflammatory, anti‐tumor and anti‐pathogen, etc. Moreover, the studies have shown that ginger can improve hemorheology events, inhibit platelet aggregation, prevent thrombosis, and play an anticoagulant effect (He et al., 2012; Jia et al., 2014). However, the understanding of its treatment of BSS is limited, and this is a problem worthy of our attention.

Metabolomic is a scientific technology for quantitative measurement of the time‐related multiparametric metabolic response of multicellular systems to pathophysiological stimuli or genetic modification (Han et al., 2016), which provides a unique chemical fingerprint of a specific organism and reveals the essence of the syndrome and the therapeutic effect of Chinese medicine; it involves “holistic‐dynamic‐comprehensive analysis” (You et al., 2009). High‐throughput metabolomics analysis has been used to reveal metabolic profiles. Owing to the massive amount of accurate chemical data, high‐speed data acquisition, and high resolution, UPLC‐Q‐TOF/MS‐based metabolomics has been widely used in dynamic analyses of biochemical changes during drug intervention, which is useful for elucidating intervention mechanisms (Chen et al., 2017; Okada & Morihito, 2012). Combined with multivariate data analysis, the metabolic profiles of each intervention group or NG can be visually displayed, and endogenous metabolites with significant differences between groups can be identified as biomarkers (Huang et al., 2020).

In this study, we established a rat model of BSS and administered different doses of dried ginger extract to the model rats. After the treatment period expired, the sample of the animal's urine, blood, and aortic vessels was collected. Pharmacodynamic indicators such as blood viscosity, aortic vascular pathology, ESR, PCV, deviation index (DI), and red blood cell accumulation index (EAI) are tested. In addition, UPLC‐Q‐TOF/MS combined with multivariate statistical analysis of serum and urine was used to conduct multi‐dimensional analysis on the metabolic spectrum and to explore the changes of its metabolites. We hope that this study can provide a new explanation for the mechanism of dried ginger in treating BSS.

## 2 MATERIALS AND METHODS

### 2.1 Materials

Methanol and acetonitrile were purchased from Dikma Technologies Inc (Beijing, USA). Ultrapure water was prepared by a Milli‐Q water purification system (Millipore, Bedford, MA, USA). The reference substance is as follows: 6‐gingerol, 8‐gingerol, 10‐gingerol, 6‐shogaol were bought from Chengdu Munster Biotechnology Co., Ltd., with 98.79%, 98.81%, 99.13%, and 98.79% purity, respectively. All the other chemicals and biochemical used are of the highest grade available. Hemorheological reagent, mass spectrometry reagent, and MS grade formic acid were purchased from Sigma‐Aldrich (St. Louis, MO, USA). The coagulometer (SYSMEXCA7000) was produced by Sysmex, Japan. PT, APTT, TT, FIB quality control plasma N.P are all Sysmex products. DG (Lot No. 201804072) was obtained from the pharmacy of the First Affiliated Hospital of Anhui University of Chinese Medicine and was identified as ginger by Professor Peng Huasheng (College of Pharmacy, Anhui University of Chinese Medicine).

### 2.2 Extraction and content identification of DG

In brief, 1.0 kg of DG was decocted for 40 min with 10 times distilled water repeatedly for two times. The decoctions were collected, mixed, filtered, concentrated under reduced pressure, and dried by vacuum at 75°C. The w/w yields of DG were 11.74%. The DG extract was re‐dissolved in distilled water to a final concentration of 2 g/ml (equivalent to the dry weight of crude drugs) before being used and further analyzed with a Waters Acquity H‐Class UPLC equipped with an Acquity BEH C_18_ column (100 mm × 2.1 mm with the particle size of 1.7 μm), which consisted of a photodiode array detector, and the mobile phase was comprised of acetonitrile (A) and water (B). The gradient mode was as follows: initial 3% A linear gradient to 85% A in 10.0 min; linear gradient to 100% A in 15.0 min; 100% B from 15.0–18.0 min, 100%–3% A over 18.0–20.0 min and final wash at 3% A over 20–22 min. The flow rate was 0.3 ml/min. The detector wavelength was set at 280 nm.

### 2.3 Animals and treatments

Male SD rats weighing 200–220 g were purchased from the Animal Center of Anhui Medical University. They were kept in plastic cages at 25 ± 2°C with free access to pellet food and water and on a 12 hr light/dark cycles. Animal welfare and experimental procedures were carried out following the guidelines for the care and use of laboratory animals (National Research Council of USA, 996). The experimental protocol was approved by the Committee on the Ethics of Animal Experiments of Anhui University of Chinese Medicine. All surgical procedures were performed under anesthesia with pentobarbital (50 mg/kg body weight), and all efforts were made to minimize suffering.

### 2.4 Experimental model and drug administration

A total of 60 SD rats were randomly divided into five groups equally, including normal group (NG), model group (MG), high dose of dried ginger group (GJH), middle dose of dried ginger group (GJM), and low dose of dried ginger group (GJL), (2.10, 1.05, 0.53 g/kg, respectively). TCM intervention groups were orally administered different doses of DG extract, respectively, and the control group was orally administered an equivalent volume of distilled water. BSS was induced by placing the rats of the model and TCM intervention groups in ice water (0–2°C) for 5 min daily for 14 consecutive days. After that, except for the NG group, other groups were injected with 0.1% adrenaline hydrochloride twice, 0.8 ml kg^−1^ each time, with an interval of 4 hr. After the first injection, rats were immersed in ice water (0–2°C) for swimming for 5 min. Rats fasted overnight, and the administration of DG extract was continued after performing the model. Blood samples were collected on the following day at 40 min after the last administration.

### 2.5 Sample collection

Urine samples were taken from six rats in each group between 18–24 hr after the second injection of epinephrine, and the rats were sodium pentobarbital anesthesia (2 ml/kg) at 24 hr after the last injection of Adr, stored at −80°C before analysis. Serum was isolated by centrifugation at 3,500 rpm for 10 min at 4°C and then frozen −80°C before metabolomics detection. Then, the other rats were anesthetized by intraperitoneal injection of pentobarbital (50 mg/kg body weight) 1 hr after the administration on the second day. The blood collection was carried out by carotid artery intubation, and the anticoagulation was carried out at the ratio of 1:9 with 3.8% sodium citrate. The whole blood viscosity, plasma viscosity, and clotting time were measured using a fully automatic hemorheometer. The blood samples of the remaining six rats in each group were drawn from the abdominal aortic to determine hemorheological variables. Blood was collected into plastic tubes with 3.8% sodium citrate for plasma anticoagulation and detected for whole blood viscosity (WBV), erythrocyte sedimentation rate blood (ESR), and packed cell volume (PCV). Then, plasma was separated from blood by centrifugation at 3,500 g for 10 min and detected for plasma viscosity (PV) and plasma anticoagulation. All experiments were completed within 4 hr after blood collection.

### 2.6 Viscosity determination

Six samples of 800 μl whole blood were taken from each group was used to determine the viscosity with a one‐plate viscometer (Model LG‐R‐80B, Steellex Co., China) at different shear rates maintained at 37°C. WBV was measured with shear rates varying from 1 to 200/S. PV was measured at the high shear rate (200/S) and low shear rate (50/S). ESR and PCV measurements are a total of 1,000 μl blood that was put into an upright Westergren tube. The rate of red blood cells falling to the bottom of the tube (mm per hour) was observed and reported. The volume of packed red blood cells was immediately measured in the tube after centrifugation (3,000 g for 30 min). Thrombin time (TT), prothrombin time (PT), activated partial thromboplastin time (APTT), and fibrinogen content (FIB) were examined with commercial kits following the manufacturer's instructions by a coagulometer (Model LG‐PABER‐I, Steellex Co., China). TT was determined by incubating 50 μl plasma solutions for 3 min at 37 °C, followed by the addition of 100 μl thrombin agent. PT was determined by incubating 50 μl plasma solutions for 3 min at 37°C, followed by the addition of 100 μl thromboplastin agents. APTT was determined by incubating 50 μl plasma with 50 μl APTT‐activating agent for 3 min at 37°C, followed by addition of 50 μl CaCl_2_. FIB was determined by incubating 10 μl plasma with 90 μl imidazole buffers for 3 min at 37°C, followed by addition of 50°C FIB agent. The anticoagulation activity was assessed by assaying the prolongation of the plasma clotting time of TT, APTT, increase INR of PT, and reduction of FIB content (Sysmex CA7000, Japan).

### 2.7 Preparation of metabolomic samples

Before analysis, six serum samples of 200 μl in each group were thawed at 4°C, and then 600 μl methanol to precipitate the proteins. Vortex the mixture for 1 min and centrifuge at 14,000 g for 15 min at 4°C. The supernatant (600 μl) was transferred to the EP tube and evaporated to dryness at 4°C under a stream of nitrogen. Dissolve the residue with 200 μl methanol followed by vortexing for 60 s and centrifuge at 14,000 rpm for 5 min. Transfer the supernatant (80 μl) to autosampler vials for UPLC‐Q‐TOF/MS analysis. The processing of urine is the same as that of serum samples. Pooling aliquots prepared a QC sample from all samples collected in the course of the study. A pooled QC sample was made by mixing aliquots (20 μl) of each sample and handled by the same method as the samples. The pooled QC sample was analyzed randomly through the analytical run to monitor instrument stability. Besides, a random sample is split into six parts and processed in the same way. These six samples were continuously analyzed injected to validate the repeatability of the sample preparation method.

### 2.8 UPLC–Q‐TOF/MS conditions

Perform serum or urine metabolic profiling on UPLC‐Q‐TOF/MS system coupled to a Waters Q‐Tof Premier Mass Spectrometer. Perform urine and serum chromatography on a Waters Acquity UPLC BEH C18 column (2.1 × 100 mm, 1.7 μm) with the temperature of the column set at 30°C. The flow rate was 0.3 ml/min‐1, and the mobile phase was ultrapure water with 0.1% formic acid (A) and acetonitrile (B). The gradient elution procedure is as follows: 0–1 min, 1% → 10% B; 1–2 min, 10% → 30% B; 2–4 min, 30% → 75% B; 4–7 min, 75% → 75% B; 7–9 min, 75% → 100% B; 9–11.5 min, 100% → 100% B; 11.5–12 min, 100% → 1% B; 12–13.5 min, 1% → 1% B. Sample analysis time was 13.5 min, and the sample injection volume was 2 μl. The autosampler maintained at 4°C. The ESI source has two working modes: positive and negative patterns. The quality test parameters are set as follows: The N_2_ flow rate is set to 650 L/h, and the positive and negative ion mode is 600 L/h, respectively. The gas temperature was 350°C. The source temperature was set to 110°C, with a cone gas flow of 100 L/hr. The capillary voltage was set at 1.5 kV in positive ion mode, and 1.8 kV in negative ion mode and the sample cone voltage was set at 100 V. All sample detections were acquired by using the lock‐spray to ensure accuracy and reproducibility. A lock‐mass at a concentration of 200 pg/ml was employed via a lock‐spray interface. The MS/MS analyses of the ions were performed at different collision energy parameters that ranged from 5 and 50 eV for plasma samples and from 10 and 50 eV for the urine samples. The ESI interface was used, and the profile data were collected in full scan mode from m/z 50–1,000. Leucine‐enkephalin was used as the lock‐mass reference compound (m/z 556.2771 in positive mode, m/z 554.2615 in negative mode) and the flow rate was 20 μl/min.

### 2.9 Data processing and analysis

The mass data acquired were imported to Markerlynx XS (Waters Corporation, MA, USA) within the Masslynx software for peak detection and alignment. The original data were processed using the following parameters: The retention time range was 0–13 min, the mass range was 50–1,000 Da, the retention time tolerance was 0.01 min, and the mass tolerance was 0.1 Da. Multivariate statistical analysis in the form of PLS‐DA and OPLS‐DA was performed using Pareto scale data. Extract potential biomarkers from S‐maps were constructed by OPLS‐DA analysis, and VIP is also used to select potential biomarkers. Variable importance in projection (VIP) score > 1 and *t* test *p*‐value < .05 were prerequisite conditions for biomarkers. PCA, OPLS‐DA, clustering heatmap analysis, correlation analysis, relative intensity analysis, and pathway analysis with MetaboAnalyst 4.0 (http://www.metaboanalyst.ca/). Other statistical analyses were performed using SPSS 22.0, and the experimental data were expressed as the mean ± *SD*. Comparisons between groups were performed using one‐way analysis of variance (ANOVA). Bilateral P values less than 0.05 were considered statistically significant.

Through the identification of biomarkers and the construction of metabolic pathways, research was conducted on essential ions found from statistical analysis to determine whether they provided potential biomarkers. These ions were tentatively identified based on their m/z value and mass spectrum using in‐house data. More specifically, the following web‐based search engines were also used to provide potential identities for these ions: Human Metabolome Database (http://www.hmdb.ca/), MassBank (http://www.massbank.jp/), and ChemSpider database (www.chemspider.com). The construction, interaction, and pathway analysis of potential biomarkers was performed with IPA (http://metpa.metabolomics.ca./MetPA/faces/Home.jsp) based on database source including the KEGG database (http://www.genome.jp/kegg/), the Human Metabolome Database (http://www.hmdb.ca/), SMPD (http://www.smpdb.ca/), and METLIN (http://metlin.scripps.edu/). The possible biological roles were evaluated by the enrichment analysis using the MetaboAnalyst, which is a web‐based tool for visualization of metabolomics.

## 3 RESULTS AND DISCUSSION

### 3.1 Content determination results of DG

**Figure 1:**
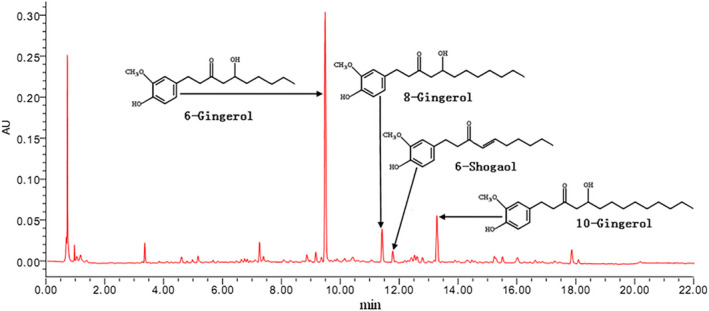
Content determination of 6‐gingerol, 8‐gingerol, and 10‐gingerol in the DG extract

The components were identified and quantified by comparison of retention time and calculation of peak areas from the chromatograms with those of known standards. The contents of 6‐gingerol, 8‐gingerol, 6‐shogaol and 10‐gingerol in DG extract were 6.95, 0.99, 0.42 and 1.32mg/g, respectively (Figure 1).

### 3.2 Pathological observation of vascular

The microstructures of the abdominal aorta in rats were observed. Vascular obstruction and a small amount of microthrombosis were observed in the MG. Some endothelial cells fell off from the vascular wall, endothelial cells swelled, and intima thickened. Also, inflammatory cell infiltration was observed. The above pathological symptoms were alleviated in the administration group, especially in the GJH (C). The results are shown in Figure S1. Histological results indicate that the vascular function of the dried ginger treatment group is protected. Concerns over the side effects and other adverse effects resulting from synthetic compounds in the treatment of BSS have been factors leading to medicinal plants as alternative choices. In view of the ability of DG extract can relax blood vessels by releasing nitric oxide and prostacyclin, activating cGMP‐KATP channels, muscarinic receptor stimulation, and transmembrane calcium channels or Ca^2+^ release from intracellular storage, it is a treatment for BSS Good drug candidate (Razali et al., 2020).

### 3.3 Effect of DG on blood viscosity

**Figure 2:**
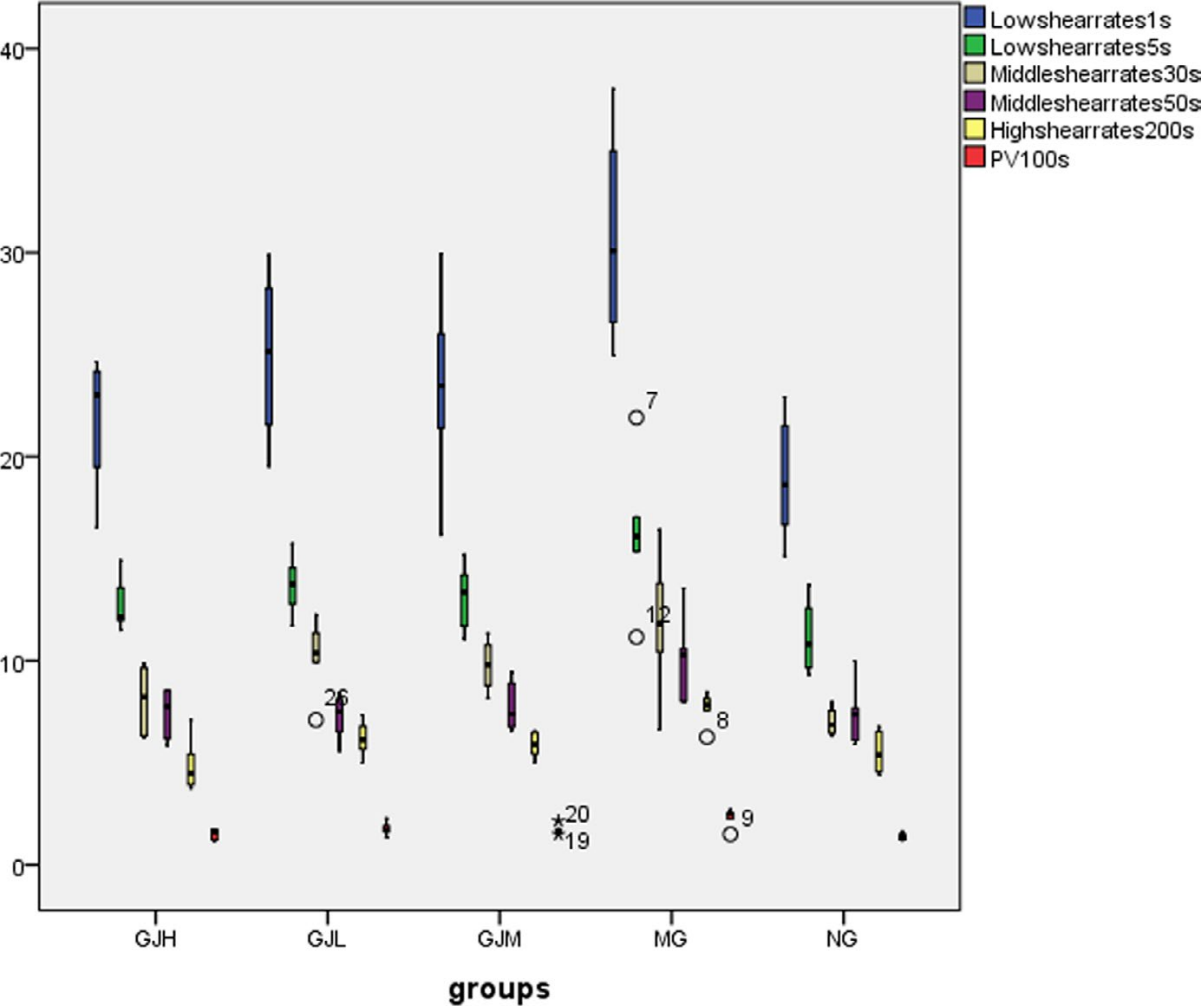
Box‐whisker plot of the effect of different shear rates on WBV (mPa•s) in each group. The WBV of the MG of BSS increased significantly at all shear rates, and the MG had a significantly higher plasma viscosity than that of each group

The impact of WBV is shown in Figure 2 and Table S1. In the MG of BSS, WBV increased significantly at all shear rates. After administration, the WBV of each group was significantly reduced at high shear rates (*p* < .01 or .05). And the table also shows the effects of DG on plasma viscosity. The model rats had a significantly higher plasma viscosity than the controls. The plasma viscosity in the GJH, GJM, GJL was significantly decreased compared to the MG (*p* < .01 or .05).

### 3.4 Effect of DG on ESR, PCV, deviation index (DI), and erythrocyte accumulation index (EAI)

The results of ESR, PCV, EAI, and DI for each group are shown in Table S2. All four indexes were significantly higher in the MG than in the NG. GJH and GJM reduced ESR and PCV (*p* < .05). All DG dose groups decreased DI and EAI (*p* < .05 or *p* < .01). It can be seen from the above that DG extract can improve blood rheology indicators.

### 3.5 Effect of DG on plasma coagulation parameters

The impacts of DG on blood coagulation were evaluated by assays of APTT, PT, TT, and FIB in the plasma. PT was decreased, FIB was increased; APTT and TT were significantly shortened in the model rats compared with the NG levels, as showed in Table S3. After administration, compared with the MG, the PT of GJH and GJM was significantly increased, and the FIB was significantly decreased (*p* < .05). In terms of TT and APTT, the DG group was significantly longer (*p* < .05). The results show that the DG extract has an effect on the coagulation system of blood stasis syndrome.

### 3.6 Metabolic profiling analysis

To obtain the maximum possible information for each metabolite, the experiments were performed in both positive and negative ionization modes and analyzed serum and urine samples under the same chromatographic conditions. The typical total ion chromatograms (TICs) of the serum and urine samples from the NG, MG, and GJH collected in the experiment are presented in Figure S2. To further analyze changes between complex sets of data, the multivariate data analysis techniques, including PCA‐X, PLS‐DA, and OPLS‐DA, were used to analyze the data.

**Figure 3:**
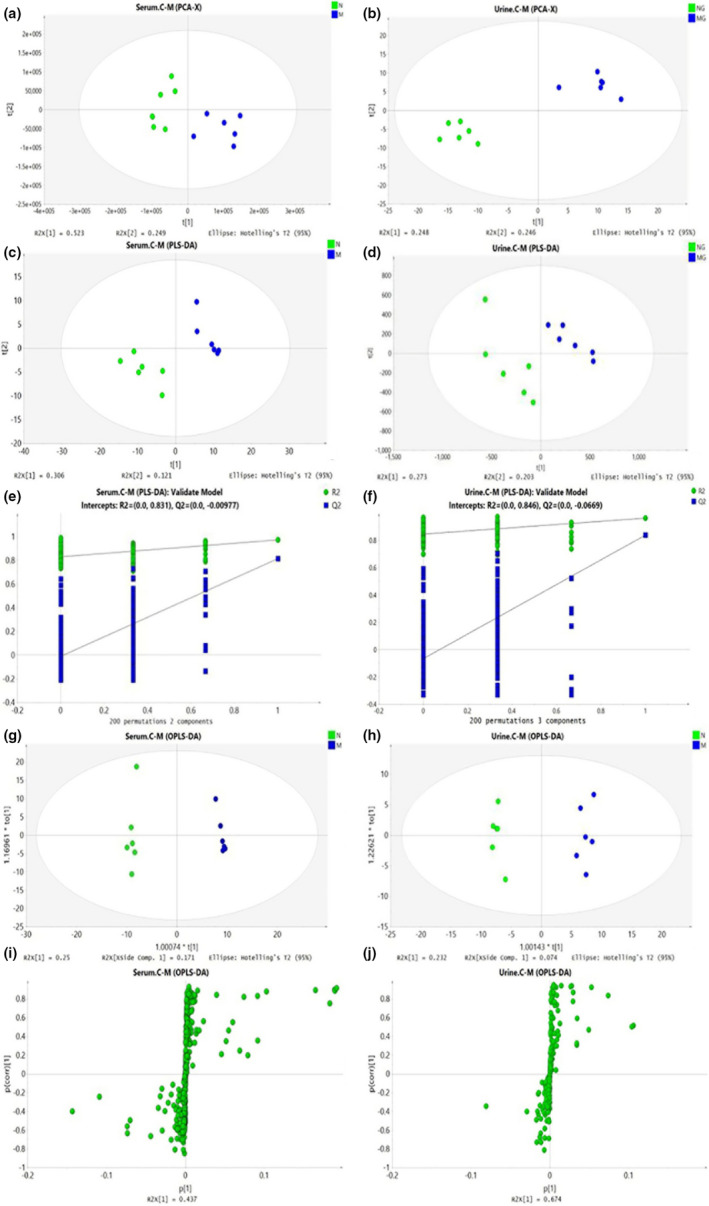
Score plots from the PCA model of the NG versus the MG for (a) serum and (b) urine and PLS‐DA model of the NG versus the MG for (c) serum and (d) urine. Two hundred permutations of the PLS‐DA model for serum (e) and urine (f). Score plots form the OPLS‐DA model of the CG versus the MG for serum (g) and urine (h). S‐Plots from the OPLS‐DA model for serum (i) and urine (j)

PCA analysis was used to assess the difference in metabolite profiles between serum and urine samples of NG and MG. The apparent separation between them was obtained in the PCA scores plot (Figure 3a,b), which indicated that the two groups had utterly different metabolic profiling. Then, the PLS‐DA method was used to systematically evaluate the metabolomics of BSS rats (permutation number: 200). In PLS‐DA, the NG was more distinct from the MG (Figure 3c,d). The PLS‐DA model parameters were as follows: *R* ^2^ = 0.846 and Q^2^ = −0.0669 in serum, and *R* ^2^ = 0.831 and Q^2^ = −0.00977 in urine, which showed an excellent predictive power (Figure 3e,f). To screen differential metabolites and maximize the discriminatory ability of serum and urine metabolites between the groups, orthogonal partial least squares discriminant analysis (OPLS‐DA) was used. As showed in the score plot (Figure 3g,h), the serum and urine samples in the MG were significantly different from those in the NG. The S‐plots (Figure 3i,j) showed differential metabolites between the two groups, and VIP was obtained based on OPLS‐DA with a threshold than 1 would be viewed as potential biomarkers. Combined with the results of the S‐plot and VIP‐value plot together, the UPLC‐Q‐TOF/MS analysis platform provided the retention time, precise molecular mass, and MS/MS data for the structural identification of biomarkers. The same procedures were utilized to analyze the plasma samples derived from the NG, GJL, GJM, and GJH.

Besides, we investigated the differences in metabolic profiles between the MG and the GJL, GJM, and GJH, using OPLS‐DA analysis. Score 3D plots (Figure S3a,b) from OPLS‐DA were used to maximize the discrimination of metabolite differences among the five groups. The figure shows that the metabolites of serum and urine in GJL, GJM, and GJH gradually approach the NG. Meanwhile, the GJH was the closest to the NG, the GJL was the closest to the MG, and the GJM was between the GJH and the GJL. Therefore, the intervention of DG on endogenous metabolites in rats with BSS shows a significant dose‐effect relationship.

### 3.7 Biomarker identification

To select potential biomarkers related to BSS, the first principal component of VIP was obtained. Firstly, the VIP values greater 1.0 are selected as changing metabolites. And then, the remaining variables were calculated by Student's *t* test (*t* test), *p* < .05, variables are discarded between two comparison groups. Besides, several commercial databases, such as HMDB (http://www.hmdb.ca), KEGG (http://www.kegg.jp), and NIST (http://www.nist.gov/index.html), were used for searching the information of metabolites.

The retention time, precise molecular mass, and MS/MS data were provided for the structural identification of potential biomarkers using the analysis platform of EZ‐info software. The accurate molecular mass was detected within 5 ppm in measurement errors by UPLC‐Q‐TOF/MS. Meanwhile, the potential elemental composition, the degree of unsaturation, and the fractional isotope abundance of compounds were obtained. The supposed molecular formula was searched in ChemSpider, the Human Metabolome Database, Mass Bank, and other relevant databases were used to identify the possible chemical constitutions, and MS/MS data were screened to determine the potential structures of the ions. Details of potential biomarkers are listed in Table 1. Metaboanalyst 4.0 (www.metaboanalyst.ca/) is used for pathway construction by employing the rice metabolic pathway databases as reference for the global test algorithm. Table 2 shows the identified biomarkers and their trends compared with the NG.

**Table 1:** Potential biomarkers in serum and urine

	NO	RT	Formula	Metabolites	VIP	*p*	FC
Serum	1	0.6595	C_3_H_4_O_3_	Pyruvate	1.0050	.0024	0.6187
	2	0.9963	C_15_H_12_O	Chalcone	2.4297	.0031	2.9408
	3	1.4248	C_29_H_50_O_4_	6‐Deoxohomodolichosterone	1.4195	.0091	2.0295
	4	2.2860	C_18_H_29_NO_2_	Penbutolol	1.2444	.0190	1.9356
	5	3.8942	C_18_H_39_NO_3_	Phytosphingosine	1.2193	.0367	1.6779
	6	6.4867	C_20_H_41_NO_3_	Ethanolamine Oleate	1.4903	.0068	2.1090
	7	7.6357	C_16_H_24_N_2_O	Oxymetazoline	1.2449	.0004	1.7684
	8	7.6965	C_19_H_38_O_4_	1‐Monopalmitin	1.6229	.0172	2.1786
	9	8.5343	C_47_H_76_O_2_	α‐calendic acid	1.0186	.0054	1.6551
	10	14.0018	C_14_H_11_NO	Phosphonoacetaldehyde	1.7977	.0004	2.2653
Urine	1	0.7043	C_14_H_11_NO	3‐Methyl‐9H‐carbazole‐9‐carboxaldehyde	1.3875	.0359	0.6445
	2	2.7173	C_22_H_32_N_2_O_5_	Benzquinamide	1.7277	.0001	0.3362
	3	0.6694	C_3_H_7_NO_2_	L‐Carnitine	1.4923	.0291	0.2745
	4	0.6896	C_4_H_9_N_3_O_2_	L‐Alanine	1.9506	.0049	0.0529
	5	0.6898	C_6_H_8_N_2_O_2_	Creatine	1.9488	.0063	0.0536
	6	0.7301	C_9_H_13_N_3_O_3_	L‐isoleucyl‐L‐proline	1.5086	.0078	0.3532
	7	1.2144	C_5_H_7_N_3_O	5‐Methylcytosine	1.3817	.0400	2.8193
	8	1.7440	C_17_H_22_N_2_O_7_	Tetrahydropentoxyline	1.5986	.0180	1.6918
	9	3.1393	C_11_H_19_NO_10_S_2_	Progoitrin	1.5839	.0082	2.6975
	10	3.1389	C_11_H_19_O_13_P	1‐Phosphatidyl‐D‐myoinositol	1.4146	.0160	2.8472
	11	9.6106	C_20_H_41_NO_3_	Ethanolamine Oleate	1.8239	.0291	2.8580
	12	9.7938	C_24_H_40_O_6_	3b,4b,7a,12a‐Tetrahydroxy‐5b‐cholanoic acid	1.8367	.0104	2.1858

**Table 2:** Summary of intensity values of potential biomarkers in each group

Matrix	Biomarkers	NG	MG	GJH	GJM	GJL
Serum	Pyruvate	2054.53 ± 219.28	3,540.26 ± 309.4^b^	2,898.62 ± 421.27	1941.29 ± 287.40^d^	2,406.15 ± 576.39^d^
	Chalcone	1,190.27 ± 311.07	49.73 ± 166.64^b^	933.49 ± 107.79	765.78 ± 125.21	655.97 ± 148.81
	6‐Deoxohomodolichosterone	1,459.10 ± 45.85	784.27 ± 132.91^b^	1,166.24 ± 315.19^c^	1,047.37 ± 371.41^c^	903.75 ± 145.43
	Penbutolol	1,379.69 ± 485.53	783.49 ± 141.84^b^	1,170.86 ± 271.58	1,066.08 ± 252.95	936.82 ± 164.42
	Phytosphingosine	29,605.10 ± 5,830.21	19,138.72 ± 4,393.29^a^	25,570.68 ± 4,553.94	23,926.42 ± 3,939.20	22,274.01 ± 4,349.25
	Ethanolamine Oleate	4,826.64 ± 1,381.75	2,523.15 ± 689.70^a^	3,780.75 ± 792.81	3,368.81 ± 72.566	2,757.25 ± 613.47
	Oxymetazoline	2,107.08 ± 130.59	1,286.33 ± 265.67^b^	1929.20 ± 245.88^d^	1657.12 ± 477.21^d^	1605.2 ± 221.02^d^
	1‐Monopalmitin	2,564.01 ± 864.06	1,280.57 ± 476.87^b^	2093.21 ± 466.46	1977.15 ± 55.80	1518.93 ± 490.59
	α‐calendic acid	965.48 ± 145.78	643.90 ± 97.28^b^	894.87 ± 103.88^c^	714.86 ± 564.18	668.67 ± 59.73
	Phosphonoacetaldehyde	7,118.85 ± 675.99	3,403.62 ± 1,038.04^b^	5,999.82 ± 1,260.77^d^	5,202.67 ± 1,139.99^c^	4,360.61 ± 1,204.03
Urine	3‐Methyl‐9H‐carbazole‐9‐carboxaldehyde	434.90 ± 136.45	728.62 ± 191.06^b^	455.14 ± 108.99^d^	448.74 ± 84.66^d^	539.19 ± 79.05^d^
	Benzquinamide	406.17 ± 153.89	1,241.39 ± 162.91^b^	583.35 ± 235.36^d^	643.73 ± 334.58^c^	717.24 ± 436.52^d^
	L‐Carnitine	619.78 ± 189.29	2,592.49 ± 1,115.51^a^	999.14 ± 250.12	1,164.39 ± 514.70	1,278.23 ± 478.98
	L‐Alanine	115.75 ± 57.41	2,866.42 ± 501.03^b^	1,011.66 ± 151.37	1,382.45 ± 603.93	1876.80 ± 939.28
	Creatine	559.21 ± 208.48	13,492.18 ± 5,090.32^b^	4,305.16 ± 1,336.07	5,777.50 ± 1,179.57	5,987.95 ± 1,430.89
	L‐isoleucyl‐L‐proline	1635.24 ± 725.09	5,166.35 ± 1748.43^a^	2,102.71 ± 1,215.84	3,787.50 ± 1,260.69	4,485.61 ± 1,489.07
	5‐Methylcytosine	3,626.66 ± 1,238.58	1,150.50 ± 609.22^b^	3,107.05 ± 944.98^d^	2,765.35 ± 1,441.79^c^	1767.78 ± 541.37^d^
	Tetrahydropentoxyline	1645.85 ± 737.43	944.80 ± 104.29^a^	1772.12 ± 655.85	1,247.53 ± 557.70	1,020.69 ± 139.07
	Progoitrin	1,257.27 ± 413.71	482.18 ± 169.51^a^	1,056.89 ± 463.63	1,132.06 ± 402.24^d^	954.89 ± 334.78^d^
	1‐Phosphatidyl‐D‐myoinositol	1,017.94 ± 622.52	355.60 ± 143.10^a^	977.23 ± 272.19^c^	961.55 ± 557.54^c^	636.25 ± 211.77
	Ethanolamine Oleate	4,809.81 ± 853.54	1997.75 ± 626.62^b^	4,761.77 ± 407.20^d^	3,986.53 ± 865.61^d^	2,888.81 ± 251.27^d^
	3b,4b,7a,12a‐Tetrahydroxy‐5b‐cholanoic acid	1,438.69 ± 259.84	735.58 ± 115.77^b^	1,484.81 ± 774.85^d^	1,343.59 ± 575.07^d^	1,164.67 ± 374.06

^a^ Compared with NG, *p* < .05.

^b^ Compared with NG, *p* < .01.

^c^ Compared with MG, *p* < .05.

^d^ Compared with MG, *p* < .01.

### 3.8 Metabolic pathway analysis

**Figure 4:**
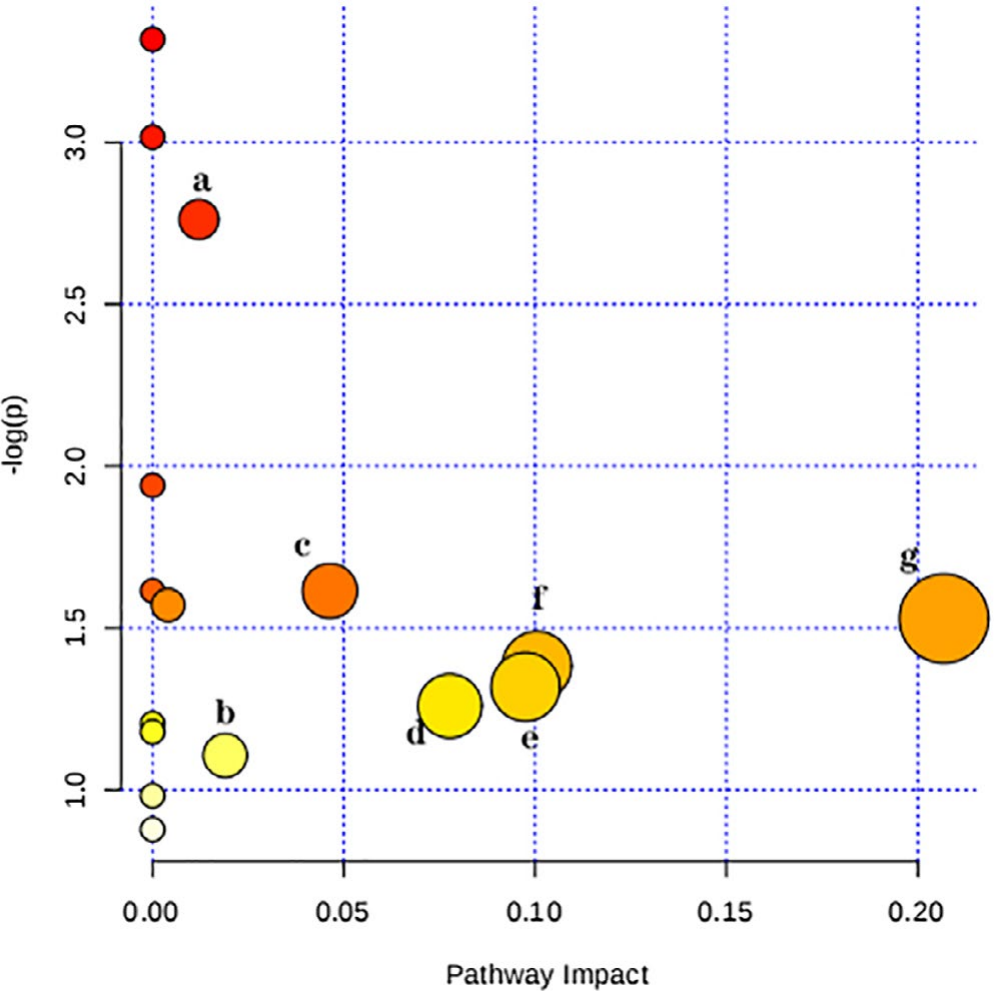
Summary of pathway analysis of serum and urine samples of the blood stasis rats. (a) Arginine and proline metabolism; (b) Glycerophospholipid metabolism; (c) Citrate cycle (TCA cycle); (d) Inositol phosphate metabolism; (e) Glycolysis/Gluconeogenesis; (f) Phosphatidylinositol signaling system; (g) Pyruvate metabolism

Possible ways to further explore the effects of BSS, using online MetaboAnalyst 4.0 software (www.metaboanalyst.ca), through the analysis of blood and urinary tract, found seven pathways affected (Figure 4) in the BSS; these ways include arginine and proline metabolism, citrate cycle (TCA cycle), pyruvate metabolism, glycolysis/gluconeogenesis, phosphatidylinositol signaling system, inositol phosphate metabolism, and glycerophospholipid metabolism. Pathways with an impact value > 0.1 were considered to be the most important pathways. The results showed that pyruvate metabolism (Impact value = 0.21) and glycolysis/gluconeogenesis (Impact value = 0.10) and phosphatidylinositol signaling system (Impact value = 0.10) were the most important pathways for the development of BSS.

Glycolysis is a common way for all organisms to extract energy from glucose, and gluconeogenesis provides a source for new glucose molecules. Glucose is produced from small carbohydrate substrates such as pyruvate, lactic acid, glycerin, and glucose amino acids, and to synthesize glucose from simple starting materials to meet the needs of various tissues (Ramirez et al., 2014; Tang et al., 2018). And in our study, when BSS occurred, the model group showed significant upregulation of pyruvate, suggesting that glucose metabolism disorders may occur, and glucose metabolism disorders indicate there appeared abnormal coagulation mechanism. After DG intervention, the pyruvate metabolism of the DG group was different from that in the model group, which indicated that the effect of DG on rats with BSS may involve the regulation of glucose metabolism.

Altered levels of myoinositol are related to inositol phosphate metabolism. Phosphatidylinositol 3‐kinases (PI3K), a key element in inositol phosphate metabolism, generates lipid second messengers that control an array of intracellular signaling pathways that play an important role in inflammation. PI3K/Akt signaling pathway can mediate the secretion of vascular endothelial growth factor (VEGF) caused by forskolin and migration of human vascular endothelial cells and the formation of new blood vessels (Namkoong et al., 2009). It has been reported that the phosphoinositol signaling pathway plays a key role in inhibiting the apoptosis of vascular endothelial cells (Chen et al., 2014). Previous studies have shown that LY294002 blocking or RNA interference with PI3K expression can inhibit the proliferation of endothelial cells and the formation of microlumen‐like structures in the area surrounding myocardial infarction in mice, accelerate myocardial cell apoptosis, increase the area of myocardial infarction area and the degree of cardiac dysfunction. This effect is mainly caused by the PI3K/Akt signaling pathway after PI3K activates Akt. When PI3K is activated and Akt is inhibited, signal transduction cannot continue, and the above effect disappears (Siragusa et al., 2010). These studies indicated the crucial role of inositol phosphate metabolism in angiogenesis. In our study, decreased myoinositol and pathway analysis suggested disruption of inositol phosphate metabolism was a characteristic of BSS, and DG extract was normalized myoinositol levels. However, the specific mechanisms of these effects have not been characterized.

Pyruvate can stimulate the transcription of fibroblast growth factor receptors and vascular endothelial growth factor m RNAs and promote the aggregation of new blood vessels in tissues. Studies have shown that pyruvate can reduce intestinal ischemia‐reperfusion injury (IRI) and effectively protect internal organs by shortening hypoxemia time, removing oxygen free radicals, inhibiting inflammatory response, increasing pH value, and increasing energy production (Zhang et al., 2020). In this study, compared with the NG, the metabolic pathway of pyruvate in the MG was destroyed, and pyruvate was abnormally increased, indicating that pyruvate level was increased under the condition of disease, and there were differences between the other groups and the MG. It indicates that the extract of dried ginger can improve the BSS by inhibiting the increase of pyruvate.

### 3.9 Correlation analysis between biomarkers and pharmacology Indicators

**Figure 5:**
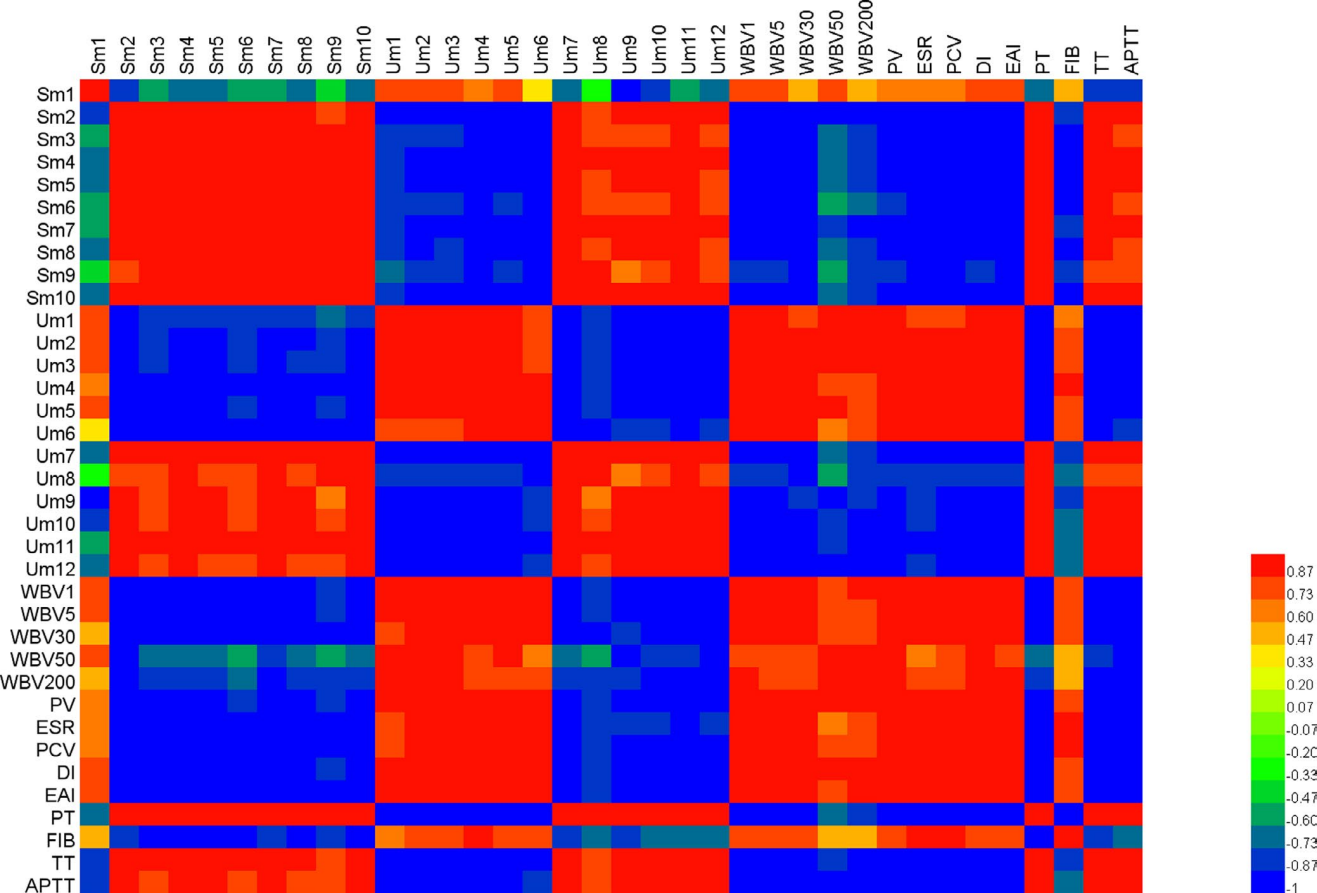
Correlation map of rat serum and urine metabolites and pharmacodynamics indices according to Pearson's correlation coefficients. Degrees of correlation degrees are shown using a color scale from significantly negatively correlated (blue) to significantly positively correlated (red)

A correlation map of rat serum and urine metabolites of biomarkers and pharmacological indicators of BSS was conducted based on Pearson's correlation coefficients. The correlation heatmap in Figure 5 shows that the metabolites of Sm1 (serum, Pyruvate), Um1 (urine,3‐Methyl‐9H‐carbazole‐9‐carboxaldehyde), Um2 (urine, Benzquinamide), Um3 (urine, L‐Carnitine), Um4 (urine, L‐Alanine), Um5 (urine, Creatine), Um6 (urine, L‐isoleucyl‐L‐proline) were positive related to the level of WBV1, WBV5, WBV30, WBV50, WBV200, PV, ESR, PCV, DI, EAI, FIB, and they are negatively correlated with PT, TT, and APTT. However, the correlation of pharmacological indicators corresponding to Sm2 (serum, Chalcone), Sm3 (serum, 6‐Deoxohomodolichosterone), Sm4 (serum, Penbutolol), Sm5 (serum, Phytosphingosine), Sm6 (serum, Ethanolamine Oleate), Sm7 (serum, Oxymetazoline), Sm8 (serum, 1‐Monopalmitin), Sm9(serum, α‐calendric acid), Sm10(serum, Phosphonoacetaldehyde) Um7 (urine, 5‐Methylcytosine), Um8 (urine, Tetrahydropentoxyline), Um9 (urine, Progoitrin), Um10 (urine, 1‐Phosphatidyl‐D‐myoinositol), Um11 (urine, Ethanolamine Oleate), Um12 (urine, 3b,4b,7a,12a‐Tetrahydroxy‐5b‐cholanoic acid) is just the opposite. Among them, Sm2 with TT, Sm8 with PT, Um4 with PCV, EAI, Um5 with DI are strongly positively correlated (*r* = .998, .998, .996, .996, .999, respectively), Sm2 with WBV1, WBV5, DI, Sm5 with PCV, Sm7 with WBV30, Um2, Um3 with APTT (*r* = −.999, −.999, −.997, −.998, −.998, −.997, −.999, respectively) are strongly negatively correlated. These correlations may indicate that changes in metabolites are related to changes in metabolites and pharmacological indicators.

### 3.10 Correlation analysis of biomarkers in each group

**Figure 6:**
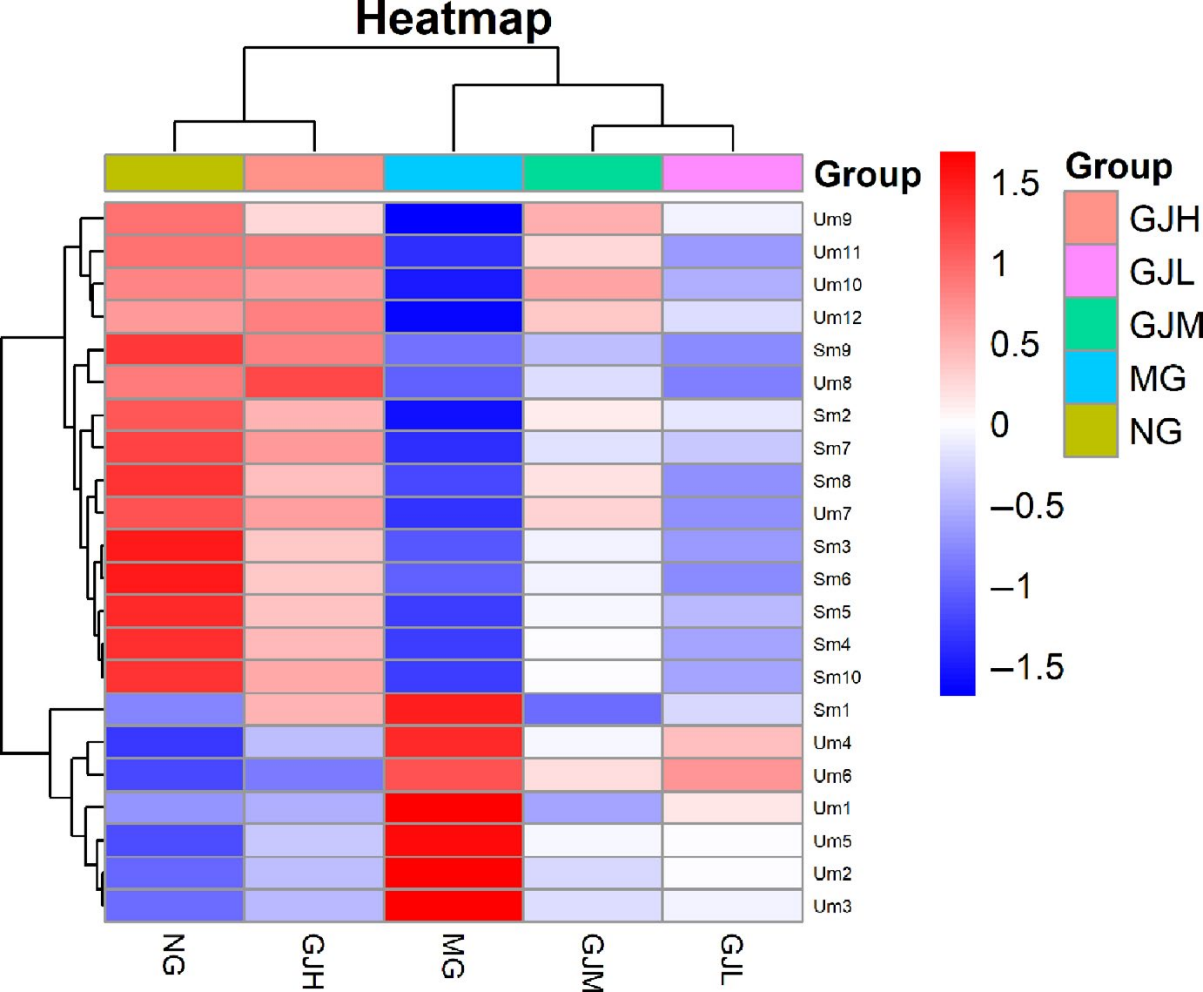
Heat map analysis of different groups of metabolites (with the deepening of red, the expression level of endogenous substances gradually increased, and with the deepening of blue, the expression level of endogenous substances gradually decreased)

The heat map (Figure 6) shows the correlation of 22 potential biomarkers in different groups. These potential biomarkers were up‐regulated and down‐regulated to different degrees in NG and MG. In addition, the results of hierarchical clustering analysis provide visual visualization of each group, the contents of metabolites Sm1(serum, Pyruvate), Um1(urine, 3‐Methyl‐9H‐carbazole‐9‐carboxaldehyde), Um2(urine, Benzquinamide), Um3(urine, L‐Carnitine), Um4(urine, L‐Alanine), Um5(urine, Creatine) and Um6 (urine, L‐isoleucyl‐L‐proline) are decreased, while the contents of other metabolites are increased, while MG and GJL were opposite to NG. It can be seen that GJH and NG metabolism are the most similar, while GJL and MG metabolism are the closest (red: increased; blue: decreased).

Due to the different contents of its components, each group had a different impact on promoting blood circulation and removing blood stasis. It is dose‐dependent. Gingerol has been reported to inhibit inflammatory factors, angiotensin II activity, platelet cyclooxygenase activity, thromboxane synthesis, and advanced glycation end products, promote myocardial sarcoplasmic reticulum Ca^2+^ ATPase activity, regulate the expression of related enzymes and proteins in the process of blood lipid metabolism, and exert cardiovascular pharmacological effects, such as cardiotonic, antiplatelet, hypolipidemic, anti‐atherosclerosis (Ying‐Zhi et al., 2017). This study can provide a scientific basis for further understanding of the mechanism of activating blood circulation and removing blood stasis by DG.

## 4 CONCLUSION

In this study, we found that DG extract can improve the pharmacodynamic indicators of blood stasis model rats, such as WBV, ESR, PCV. UPLC‐Q‐TOF/MS metabolomics and multivariate statistical analysis were used to compare the efficacy of different doses of DG extract on BSS. Through the research of metabolites in rats with BSS, it was found that 22 metabolites in rats with BSS had significant metabolic differences, which may be potential biomarkers or therapeutic targets in the development of BSS. The effect of DG on BSS may be related to the regulation of glycolysis/ gluconeogenesis, phosphatidylinositol signaling system, and pyruvate metabolism. In addition, we found that DG has a dose‐dependent increase or decrease in the metabolism of BSS; these findings reveal the possible mechanism of action of DG in the treatment of BSS. Therefore, this study also provides a basis for exploring DG as an alternative drug for treating blood stasis syndrome; however, the specific mechanism of pharmacodynamics is not fully clear, and future studies are needed to investigate the potential roles of DG in the regulation of the selected endogenous metabolites associated with BSS.

## CONFLICTS OF INTEREST

The authors declare no conflict of interest.

## AUTHOR CONTRIBUTIONS

Min Su carried out the main experiments and drafted this manuscript. Gang Cao and Daniel Raftery participated in metabonomics research and data analysis. Xiaoli Wang completed the determination and analysis of UPLC. Yan Hong designed part of the efficacy experiment and revised the manuscript. Yanquan Han directed the experimental research and manuscript writing. All authors read and approved the final manuscript.

## ETHICAL APPROVAL

The experimental protocol was approved by the Committee on the Ethics of Animal Experiments of Anhui University of Chinese Medicine (Anhui, China; permit number: 2019AH‐038‐04).

## Supporting information

Supinfo

## Data Availability

The data that supports the findings of this study are available in the supplementary material of this article.

## References

[fsn31929-bib-0001] Brahe, L. K. , Arne, A. , & Larsen, L. H. (2016). Can we prevent obesity‐related metabolic diseases by dietary modulation of the gut microbiota? Advances in Nutrition. 7, 90–101. 26773017 10.3945/an.115.010587 PMC4717895

[fsn31929-bib-0002] Cao, D. , Xu, C. , Xue, Y. , Ruan, Q. , Yang, B. , Liu, Z. , & Jin, J. (2018). The therapeutic effect of Ilex pubescens extract on blood stasis model rats according to serum metabolomics. Journal of Ethnopharmacology, 227, 18–28. 30142425 10.1016/j.jep.2018.08.026

[fsn31929-bib-0003] Chen, E. , Lu, J. , Chen, D. , Zhu, D. , Wang, Y. , Zhang, Y. , & Li, L. (2017). Dynamic changes of plasma metabolites in pigs with GalN‐induced acute liver failure using GC–MS and UPLC–MS. Biomedicine & Pharmacotherapy, 93, 480–489. 28668767 10.1016/j.biopha.2017.06.049

[fsn31929-bib-0004] Chen, W. Q. , Huang, X. B. , Wang, N. Q. , & Chen, Y. J. (2014). Effect of paired using tangerine peel and ternate pinellia tuber on the expressions of phosphatidylinositol 3‐kinase and phosphorylation of protein kinase B/Akt in rabbits with carotid atherosclerosis. Chinese Journal of Cerebrovascular Diseases, 11(7), 364–367.

[fsn31929-bib-0005] Ezzat, S. M. , Ezzat, M. I. , Okba, M. M. , Menze, E. T. , & Abdel‐Naim, A. B. (2018). The hidden mechanism beyond ginger ( Zingiber officinale Rosc.) potent in vivo and in vitro anti‐inflammatory activity. Journal of Ethnopharmacology, 214, 113–123. 29253614 10.1016/j.jep.2017.12.019

[fsn31929-bib-0006] Fang, W. T. , Zhan, Z. L. , Peng, H. S. , & Huang, L. Q. (2017). Historical evolution and change of differentiation on dried ginger, fresh ginger and baked ginger. China Journal of Chinese Materia Medica, 42(9), 1641–1645. 29082683 10.19540/j.cnki.cjcmm.2017.0065

[fsn31929-bib-0007] Gunathilake, K. , & Rupasinghe, H. V. (2015). Recent perspectives on the medicinal potential of ginger.

[fsn31929-bib-0008] Han, Y. , Li, Y. , Wang, Y. , Gao, J. , Xia, L. , & Hong, Y. (2016). Comparison of fresh, dried and stir‐frying gingers in decoction with blood stasis syndrome in rats based on a GC–TOF/MS metabolomics approach. Journal of Pharmaceutical and Biomedical Analysis, 129, 339–349. 27454085 10.1016/j.jpba.2016.07.021

[fsn31929-bib-0009] He, S. , Rong, Z. , Wei, Z. , Li, X. , Li, W. , Mi, S. , & Wang, N. (2012). Effects of Pericarpium Citri Reticulatae and Rhizoma Zingiberis extracts on hemarheology and platelet aggregation of blood stasis model rats. Pharmacology & Clinics of Chinese Materia Medica.28, 11–13.

[fsn31929-bib-0010] Huang, H. , Wu, J. , Lu, R. , Liu, X. , Chin, B. , Zhu, H. , & Su, Z. (2020). Dynamic urinary metabolomics analysis based on UHPLC‐Q‐TOF/MS to investigate the potential biomarkers of blood stasis syndrome and the effects of Danggui Sini decoction. Journal of Pharmaceutical and Biomedical Analysis, 179, 112986 10.1016/j.jpba.2019.112986 31787459

[fsn31929-bib-0011] Jatoi, S. A. , Kikuchi, A. , Gilani, S. A. , & Watanabe, K. N. (2007). Phytochemical, pharmacological and ethnobotanical studies in mango ginger ( Curcuma amada Roxb.; Zingiberaceae). Phytotherapy Research, 21(6), 507–516. 10.1002/ptr.2137 17397131

[fsn31929-bib-0012] Jia, D. B. , Miao, Y. U. , Nai‐Min, L. I. , Tang, L. M. , Zhu, Y. , Chun‐Jie, L. I. , & Pla, H. O. (2014). Effects of Shenjiang suoyang yiqi tablets on blood rheology and vascular endothelial function in blood stasis due to cold accumulation and stagnation of the circulation of vital energy model rats. Lishizhen Medicine & Materia Medica Research, 10, 2347–2348.

[fsn31929-bib-0013] Jian, W. X. , Chen, Q. H. , & Huang, X. P. (2012). Metabolomics study of the myocardial tissue of rats of cardiac blood stasis syndrome. Zhongguo Zhong Xi Yi Jie He Za Zhi Zhongguo Zhongxiyi Jiehe Zazhi = Chinese journal of integrated traditional and Western medicine / Zhongguo Zhong xi yi jie he xue hui, Zhongguo Zhong yi yan jiu yuan zhu ban, 32(4), 515–520. 22803435

[fsn31929-bib-0014] Kelly, B. B. , Narula, J. , & Fuster, V. (2012). Recognizing global burden of cardiovascular disease and related chronic diseases. Mount Sinai Journal of Medicine A Journal of Translational & Personalized Medicine, 79(6), 632–640. 10.1002/msj.21345 23239202

[fsn31929-bib-0015] Lee J. O. , Kim N. , Lee H. J. , Moon J. W. , Lee S. K. , Kim S. J. , … Kim H. S. (2015). [6]‐Gingerol affects glucose metabolism by dual regulation via the AMPKα2‐mediated AS160‐Rab5 pathway and AMPK‐mediated insulin sensitizing effects. Journal of Cellular Biochemistry, 116, 1401–1410. 25694332 10.1002/jcb.25100

[fsn31929-bib-0016] Lee, S. H. , Cekanova, M. , & Baek, S. J. (2008). Multiple mechanisms are involved in 6‐gingerol‐induced cell growth arrest and apoptosis in human colorectal cancer cells. Molecular Carcinogenesis, John Wiley & Sons Ltd, 47(3), 197–208. 10.1002/mc.20374 18058799 PMC2430145

[fsn31929-bib-0017] Liu, H. , Zhang, W.‐J. , Long, C.‐F. , & Su, W.‐W. (2017). Protective effects of traditional Chinese herbal formula Compound Xueshuantong Capsule (CXC) on rats with blood circulation disorders. Biotechnology & Biotechnological Equipment, 31(4), 1–9. 10.1080/13102818.2017.1301785

[fsn31929-bib-0018] Ma, X. J. , Yin, H. J. , & Chen, K. J. (2009). Differential gene expression profiles in coronary heart disease patients of blood stasis syndrome in traditional Chinese medicine and clinical role of target gene. Chinese Journal of Integrative Medicine, 15(2), 101–106. 10.1007/s11655-009-0101-4 19407946

[fsn31929-bib-0019] Namkoong, S. , Kim, C. K. , Cho, Y. L. , Kim, J. H. , Lee, H. , Ha, K. S. , & Kwon, Y. G. (2009). Forskolin increases angiogenesis through the coordinated cross‐talk of PKA‐dependent VEGF expression and Epac‐mediated PI3K/Akt/eNOS signaling. Cellular Signalling, 21(6), 906–915. 10.1016/j.cellsig.2009.01.038 19385062

[fsn31929-bib-0020] Okada, & Morihito (2012). Metabolomic analysis of dynamic response and drug resistance of gastric cancer cells to 5‐fluorouracil. Oncology Reports, 29, 925–931. 23232983 10.3892/or.2012.2182 PMC3597557

[fsn31929-bib-0021] Peng, F. , Tao, Q. , Wu, X. , Dou, H. , Spencer, S. , Mang, C. , & Hao, X. (2012). Cytotoxic, cytoprotective and antioxidant effects of isolated phenolic compounds from fresh ginger. Fitoterapia, 83(3), 568–585. 10.1016/j.fitote.2011.12.028 22248534

[fsn31929-bib-0022] Ramirez, J. , Periyakaruppan, A. , Sarkar, S. , Ramesh, G. T. , & Sharma, S. C. (2014). Effect of simulated microgravity on the activity of regulatory enzymes of glycolysis and gluconeogenesis in mice liver. Microgravity Science & Technology, 25(5), 303–309. 10.1007/s12217-013-9356-7

[fsn31929-bib-0023] Razali, N. , Dewa, A. , Asmawi, M. Z. , Mohamed, N. , & Manshor, N. M. (2020). Mechanisms underlying the vascular relaxation activities of Zingiber officinale var. rubrum in thoracic aorta of spontaneously hypertensive rats. Journal of Integrative Medicine, 18(01), 46–58. 31882255 10.1016/j.joim.2019.12.003

[fsn31929-bib-0024] Shareef, H. K. , Muhammed, H. J. , Hussein, H. M. , Hameed, I. H. (2016). Antibacterial effect of ginger (Zingiber officinale) roscoe and bioactive chemical analysis using gas chromatography mass spectrum. Oriental Journal of Chemistry an International Research Journal of Pure & Applied Chemistry, 32, 817–837.

[fsn31929-bib-0025] Singh, G. , Kapoor, I. , Singh, P. , de Heluani, C. S. , de Lampasona, M. P. , & Catalan, C. A. N. (2008). Chemistry, antioxidant and antimicrobial investigations on essential oil and oleoresins of Zingiber officinale. Food and Chemical Toxicology, 46(10), 3295–3302. 10.1016/j.fct.2008.07.017 18706468

[fsn31929-bib-0026] Siragusa, M. , Katare, R. , Meloni, M. , Damilano, F. , Hirsch, E. , Emanueli, C. , & Madeddu, P. (2010). Involvement of phosphoinositide 3‐kinase ? in angiogenesis and healing of experimental myocardial infarction in mice. Circulation Research, 106(4), 757–768. 20056919 10.1161/CIRCRESAHA.109.207449 PMC2833289

[fsn31929-bib-0027] Tang, Y. , Zhang, Y. , Wang, C. , Sun, Z. , Li, L. , Cheng, S. , & Zhou, W. (2018). Overexpression of PCK1 gene antagonizes hepatocellular carcinoma through the activation of gluconeogenesis and suppression of glycolysis pathways. Cellular Physiology & Biochemistry, 47(1), 344–355. 10.1159/000489811 29768256

[fsn31929-bib-0028] Tohma, H. , Gülçin, İ. , Bursal, E. , Gören, A. C. , & Köksal, E. (2017). Antioxidant activity and phenolic compounds of ginger (Zingiber officinale Rosc.) determined by HPLC‐MS/MS. Journal of Food Measurement & Characterization, 11, 556–566.

[fsn31929-bib-0029] Xiao‐yan, L. , Hao, X. , Tie, Z. , & Geng, L. (2018). Study of serum metabonomics and formula‐pattern correspondence in coronary heart disease patients diagnosed as phlegm or blood stasis pattern based on ultra performance liquid chromatography mass spectrometry. Chinese Journal of Integrative Medicine, 24(12), 905–911. 10.1007/s11655-018-2564-7 29948595

[fsn31929-bib-0030] Ying‐Zhi, W. U. , Qiang, F. U. , Yan, Q. N. , Zhi‐Liang, L. I. , Cardiology, D. O. , Hospital, Z. , & University, S. M. (2017). Research progress on pharmacological actions of gingerols in cardiovasular disease. Chinese Journal of Clinical Pharmacology, 18, 1824–1827.

[fsn31929-bib-0031] You, W. H. , Wang, P. , Mao‐Qing, L. I. , Zhang, Y. , & Peng, Y. L. (2009). Therapeutic effects of modified Danggui Sini Decoction on plasma level of advanced glycation end products in patients with Wagner grade 0 diabetic foot:A randomized controlled trial. Zhong XI Yi Jie He Xue Bao, 7(7), 622–628. 10.3736/jcim20090705 19615315

[fsn31929-bib-0032] Zhang, J.‐J. , Deng, J.‐T. , Shen, H.‐Q. , Jiang, L.‐L. , He, Q.‐W. , Zhan, J. , & Wang, Y.‐L. (2020). Pyruvate protects against intestinal injury by inhibiting the JAK/STAT signaling pathway in rats with hemorrhagic shock. Journal of Surgical Research, 248, 98–108. 10.1016/j.jss.2019.11.012 31877436

