# Supplementary material for: Metabolomics study of dried ginger extract on serum and urine in blood stasis rats based on UPLC‐Q‐TOF/MS
Source: Food Sci Nutr. 2020 Oct 5;8(12):6401–14. doi: 10.1002/fsn3.1929 (PMC7723213; doi:10.1002/fsn3.1929)
Supplement: Supplementary file 1 — Supinfo [file FSN3-8-6401-s001.doc]

Table S1. Effects of different shear rates on WBV (mPa · s) in each group

| Group | Dose | Whole blood viscosity | | | | | Plasma viscosity  （/100s） |
| --- | --- | --- | --- | --- | --- | --- | --- |
|  |  | High shear rates（/200s） | Middle shear rates（/50s） | Middle shear rates（/30s） | Low shear rates(/5s) | Low shear rates  (/1s) |  |
| NG | — | 5.52±0.99 | 7.41±1.46 | 7.01±0.69 | 11.16±1.74 | 18.91±2.94 | 1.39±0.18 |
| MG | — | 7.69±0.76^##^ | 10.12±2.07^#^ | 11.82±3.31^##^ | 16.28±3.45^##^ | 30.78±4.96^##^ | 2.33±0.43^##^ |
| GJH |  | 4.86±1.24^**^ | 7.44±1.16^*^ | 8.09±1.58^*^ | 12.71±1.29^*^ | 21.82±3.21^**^ | 1.51±0.25** |
| GJM |  | 5.88±0.60^**^ | 7.75±1.17 | 9.78±1.23 | 13.15±1.57^*^ | 23.41±4.70^*^ | 1.74±0.22* |
| GJL |  | 6.18±0.87^*^ | 7.28±1.06^*^ | 10.24±1.76 | 13.72±1.42 | 24.92±4.00 | 1.76±0.31* |

Note. Data represent mean±S.D. n=6. #Compared with NG, p < 0.05. ##Compared with NG, p < 0.01. *Compared with MG, p < 0.05. **Compared with MG, p < 0.01.

Table S2 Effect of DG extract on ESR, PCV, DI, and EAI

| Group | Dose(g/kg) | ESR（MM/H） | PCV(%) | DI (%) | EAI |
| --- | --- | --- | --- | --- | --- |
| NG | — | 3.30±1.15 | 31.67±7.34 | 66.17±7.63 | 2.23±0.40 |
| MG | — | 10.37±2.85^##^ | 51.83±7.70^##^ | 83.83±7.65^##^ | 5.24±0.92^##^ |
| GJH | 2.10 | 6.28±1.06* | 39.17±8.11* | 71.33±7.39* | 3.08±0.65** |
| GJM | 1.05 | 7.68±0.43* | 42.33±6.09* | 73.17±8.04* | 3.49±0.88** |
| GJL | 0.53 | 7.90±1.64 | 44.50±6.89 | 74.33±6.62* | 3.92±0.83* |

Note. Data represent mean±S.D. n=6. #Compared with NG, p < 0.05. ##Compared with NG, p < 0.01. *Compared with MG, p < 0.05. **Compared with MG, p < 0.01.

Table S3 Effect of DG extract on Plasma Coagulation Parameters

| Group | Dose(g/kg) | PT(INR) | FIB (g) | TT(S) | APTT(S) |
| --- | --- | --- | --- | --- | --- |
| NG | — | 1.37±0.15 | 2.11±0.26 | 48.90±4.70 | 32.33±5.75 |
| MG | — | 0.88±0.21^##^ | 6.38±0.79^##^ | 30.77±6.30^##^ | 20.67±4.41^##^ |
| GJH | 2.10 | 1.21±0.16* | 5.16±1.02* | 44.24±5.42** | 30.50±8.55* |
| GJM | 1.05 | 1.16±0.21* | 5.33±0.803* | 42.53±645* | 29.33±7.39* |
| GJL | 0.53 | 1.00±0.18 | 5.66±1.08 | 39.82±5.44* | 28.67±7.01* |

Note. Data represent mean±S.D. n=6. #Compared with NG, p < 0.05. ##Compared with NG, p < 0.01. *Compared with MG, p < 0.05. **Compared with MG, p < 0.01.


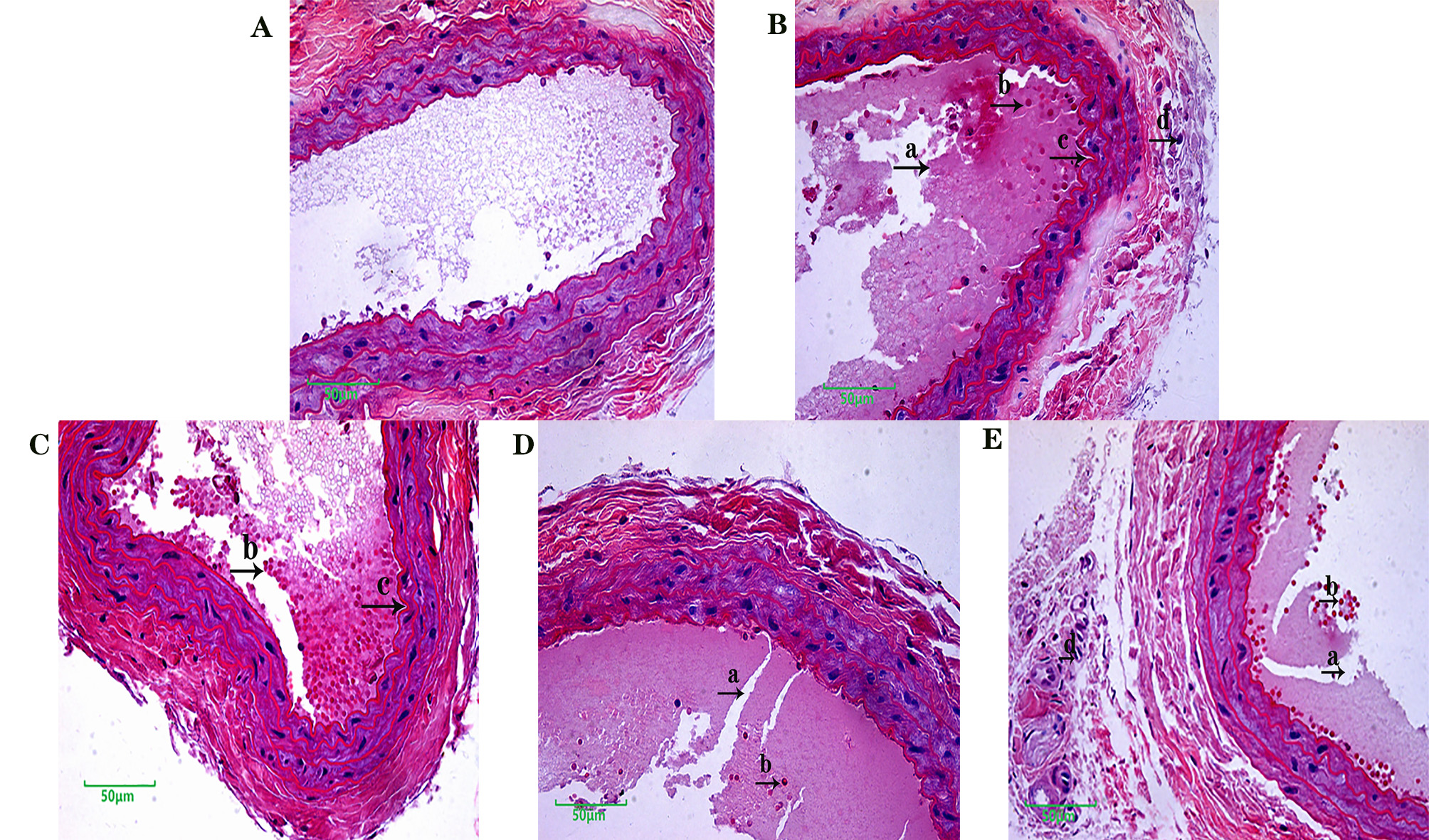


Figure S1 Histopathology (with H&E staining) of rats’ abdominal aorta of NG (A), MG (B), GJH (C), GJM (D), and GJL (E) The vessel wall and vascular endothelial cells. (H&E, 400×). a.thickening of the intima, b. cellular debris, c.vascular occlusion, d. inflammatory cell infiltration.


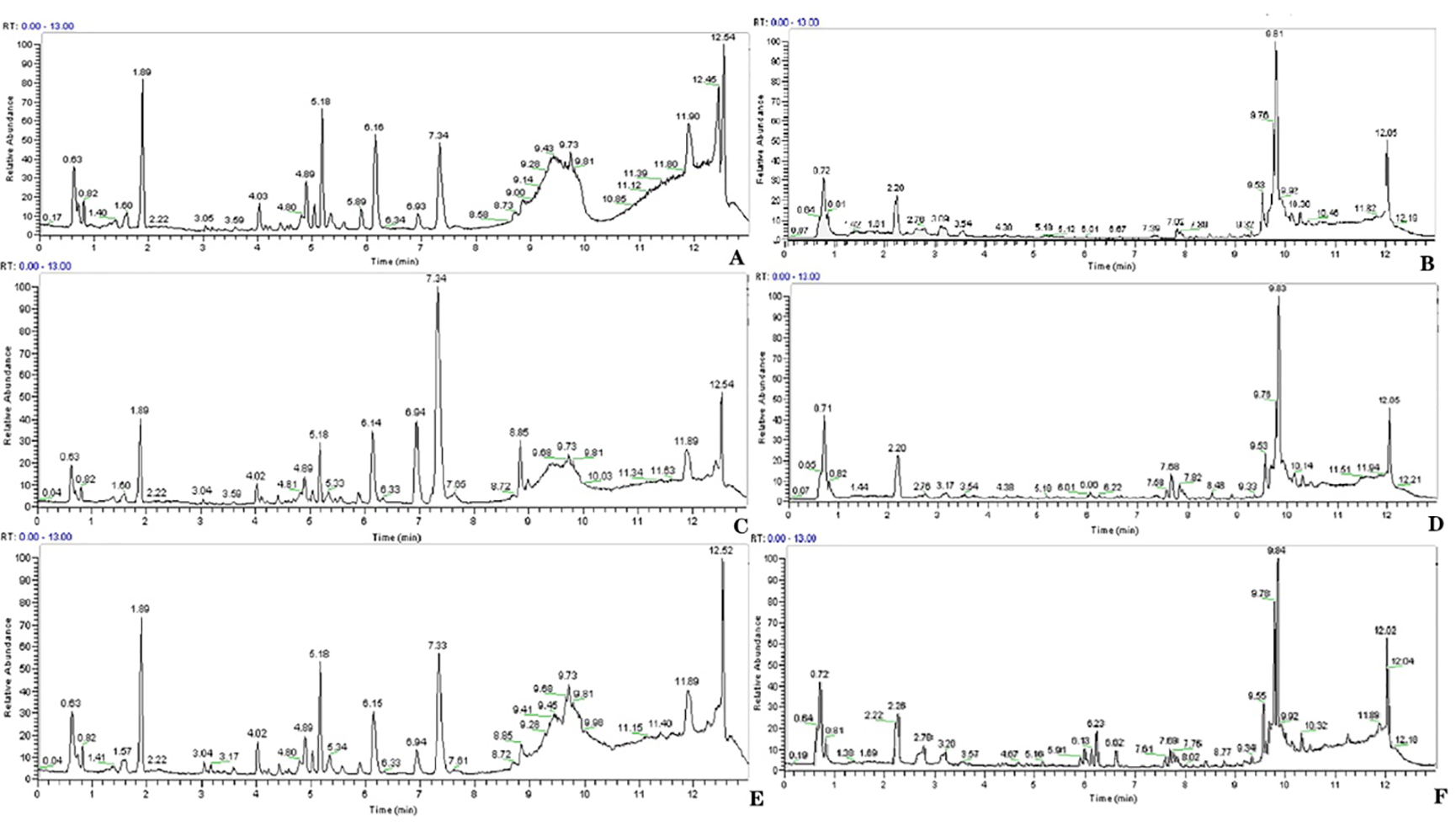


Figure S2 The Representative TIC chromatograms in positive ion mode of plasma (A-C) and urine (D-F) samples derived from NG（A, D）, MG (B, E)and GJH (C, F).


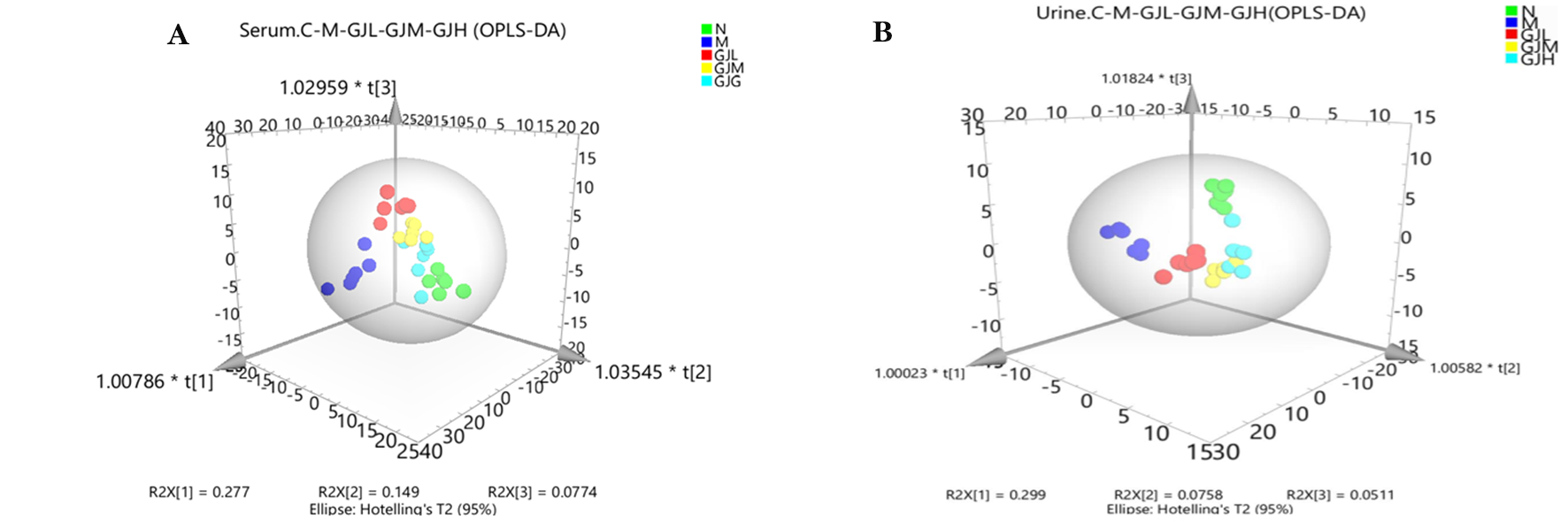


Figure S3 OPLS-DA scores 3D plots.
